# Macro-vacuolar steatosis in a cirrhotic liver mimicking metastatic disease

**DOI:** 10.1016/j.radcr.2024.11.025

**Published:** 2024-12-03

**Authors:** Pietro Pitrone, Agatino Cacciola, Antonino Cattafi, Alessia Maria Romeo, Annalisa Cracò, Francesco Marcello Aricò, Nicola Migliaccio, Francesca Magnani, Italo Giuseppe Bellone, Simona Caloggero, Giampiero Mastroeni

**Affiliations:** a Radiology Unit, "Papardo" Hospital, Messina, ME 98158, Italy; b Diagnostic and Interventional Radiology Unit, BIOMORF Department, University Hospital "Policlinico G. Martino", Messina, ME 98124, Italy; c Provincial Health Agency of Messina, ME, Italy; d Provincial Health Agency of Reggio Calabria, RC, Italy

**Keywords:** Multinodular steatosis, Cirrhosis, Metastatic disease, Core biopsy

## Abstract

Multinodular steatosis represents a relatively uncommon manifestation of fatty liver disease (FLD). Co-morbidities such as metabolic syndrome or cirrhosis are often associated. Despite typical features of imaging (ultrasound, CT, and MRI), core biopsy sometimes remains the gold standard for diagnosis. We describe the case of a 57-year-old male patient with a long history of hepatic cirrhosis and a recent diagnosis of carcinoma of the tongue, successfully treated. Due to the occurrence of nausea, diarrhea and jaundice the patient is admitted to Our Hospital where ultrasound examination and contrast-enhanced CT are performed, showing global hypoechogenicity of the liver parenchyma with multiple hypo-attenuating lesions. To rule out metastatic lesions, contrast-enhanced CT of the thorax and cranium and gastroscopy and colonoscopy are performed, with no evidence of primary malignancy. Core biopsy reveals macro-vacuolar steatosis within a cirrhotic liver with regenerative aspects.

## Introduction

Fatty liver disease shows a high prevalence (15%-25%) within the general population and might have a benign course (as in the case of simple steatosis) or, whenever inflammation occurs, lead to fibrosis and cirrhosis. When dealing with steatosis, the most frequent patterns are "focal" and "diffuse"; however, a multinodular or "patchy" appearance is reported and may mimic other pathologies, such as metastatic disease. History taking (eg, alcohol consumption, cirrhosis), laboratory tests (such as liver enzymes and tumor markers) and imaging (ultrasound, CT, MRI) are relevant in making a diagnosis; nevertheless, core biopsy often remains the gold standard.

## Case report

**Fig. 1:**
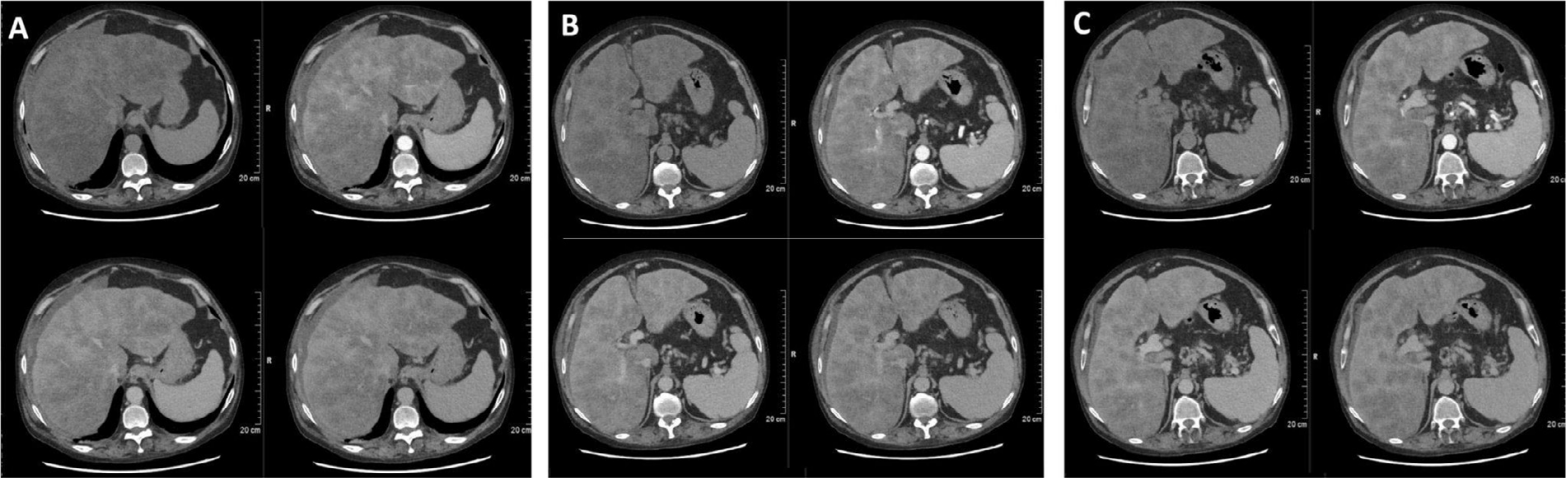
unenhanced and contrast-enhanced CT axial scans at different levels (A-C) showing multiple hypo-attenuating lesions spread throughout the liver, which appears hypertrophic with irregular margins.

**Fig. 2:**
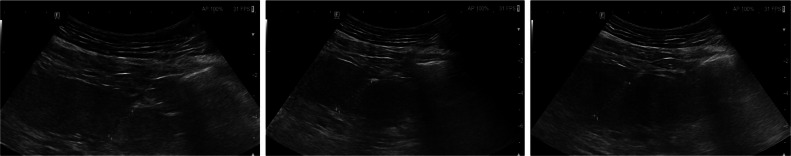
ultrasound transverse scans of the left lobe showing diffuse and heterogeneous hypoechogenicity of liver parenchyma.

A 57-year-old male patient with a long history of tobacco and alcohol consumption and hepatic cirrhosis with esophagus varices (F1-GOV1), hypertensive gastropathy and encephalopathy, is diagnosed with squamocellular carcinoma of the tongue with lateral-cervical metastases (pT3N3b cM0; month 0) and undergoes surgery and adjuvant radiotherapy and chemotherapy between months 6 and 9. No recurrence is observed at the oncologic follow-up at 12 months. Due to the occurrence of nausea, diarrhea and mild jaundice, the patient is admitted to Our Hospital where laboratory tests show low platelets (62 × 10^3/uL; n.v.: 150-400 × 10^3/uL) and high serum levels of bilirubin (total 11 mg/dL [n.v.: 0.1-1.3 mg/dL], indirect 2.9 mg/mL [n.v.: 0-1 mg/dL], direct 8.1 mg/dL [n.v.: 0-0.3 mg/dL]), GOT/AST (281 U/L; n.v.: 14-36 U/L), GPT/ALT (119 U/L; n.v.: 0-35 U/L), GT (908 U/L; n.v.: 12-43 U/L), LDH (376 U/L; n.v.: 84-246 U/L), ALP (208 U/L; n.v.: 44-147 U/L), CEA (6.4 ng/mL; n.v.: 0-3 ng/mL), CA-125 (469 U/mL; n.v.: 0-35 ng/mL), CA 15-3 (49 U/mL; n.v.: 0-35 ng/mL), CA 19-9 (841 U/mL; n.v.: 0-37 ng/mL). A contrast-enhanced CT shows multiple hypo-attenuating lesions spread throughout the liver (Fig. 1), moderate splenomegaly, dilatation of the porta, the splenic and the mesenteric vein, recanalization of the para-umbilical veins and aspecific hepatic hilar nodes. On ultrasound examination, the liver appears diffusely and heterogeneously hypoechoic (Fig. 2). To rule out secondary lesions, contrast-enhanced CT of the thorax and skull and gastroscopy and colonoscopy are performed, with no evidence of primary tumors. Core biopsy is thus performed: surprisingly, only macro-vacuolar steatosis within a cirrhotic liver with regenerative aspects is demonstrated.

## Discussion

Fatty liver disease affects about 15%-25% of the general population and occurs due to excessive lipid accumulation (triglycerides and other cholesterol derivates) within the liver cells, typically around the central veins (hypoxic areas). Simple steatosis (as in NAFLD) usually shows a relatively benign course; however, inflammation (nonalcoholic steatohepatitis: NASH) may occur, eventually leading to fibrosis, cirrhosis, liver failure, hepatocellular carcinoma and death [1,2]. Besides "focal" and "diffuse" patterns (the latter being the most frequent and typical), hepatic steatosis might show a multinodular or "patchy" appearance, thus resembling metastasis or pseudo-tumors (such as inflammatory or vascular pathologies) [3,4]. Most patients are asymptomatic and the disease is detected randomly during laboratory or instrumental examinations; some may present with manifestations of metabolic syndrome such as visceral obesity, dyslipidemia, impaired glucose metabolism and arterial hypertension [5]. FLD is associated with specific imaging features: ultrasonography shows increased echogenicity within the parenchyma with beam attenuation due to fatty infiltration; CT demonstrates hypodensity, absence of mass effect and regular course of the vascular structures; finally, MRI proves a typical drop of signal on out-of-phase gradient echo images due to intracellular lipid content (chemical shift) and absence of contrast enhancement or diffusion restriction [6]. Nevertheless, there is evidence of limited sensitivity and specificity of CT without (33% and 100%) and with contrast administration (50% and 83%) and MRI (88% and 63%) [5]; thus, histological examination still represents the gold standard for diagnosis [7,8]. Despite invasiveness, high costs, limited study areas, potential unsatisfactory samples or complications, histology can confirm the presence of FLD, distinguish between steatosis and steatohepatitis and evaluate the stage of concomitant fibrosis [6]. Similar cases are already described in the literature. P. Burko et al. [6] reported the case of an asymptomatic 34-year-old male patient with multiple hypodense nodules, reduced elasticity on shear wave elastography (SWE) and the above-described features in MRI, with histological diagnosis of steatohepatitis (second degree) and fibrosis (first stage). H.Q. Duong et al. [9] reported the case of a 50-year-old male patient with only mild elevation of serum cholesterol (6.98 mmol/L; n.v.:) and triglycerides (2.59 mmol/L; n.v.:) and incidental finding of multiple liver nodules, isoechoic with a clear hyperecoic rim, appearing hypoattenuating on unenhanced CT-scans with the typical drop in signal intensity in out-of-phase MRI scans. After nondiagnostic colo-rectal and gastro-intestinal endoscopies, a core biopsy revealed the presence of multifocal fatty liver nodules. Finally, E.K. Fonseca et al. [10] reported the case of a 56-year-old male with a chondroid chordoma at clivus and multiple hypoattenuating nodules within the liver, more evident on unenhanced scans than enhanced, as well as in MRI. Our patient showed significative laboratory alterations (including high serum levels of CEA, CA-125, CA 15-3 and CA 19-9) and cirrhotic hepatomegaly with global hypoecogenicity (differently from the above-described cases) and multiple hypo-attenuating lesions on CT. The concern for metastatic lesions led us to perform a core biopsy rather than MRI (potentially non-diagnostic) or simple follow-up.

## Conclusion

Multinodular or "patchy" steatosis may mimic metastatic liver disease, especially in patients with hepatopathy (such as cirrhosis) associated with global parenchymal hypoecogenicity. Despite the usual co-morbidities (such as manifestations of metabolic syndrome and liver enzyme alterations) and the typical imaging features (on ultrasound, CT and MRI), core biopsy often remains the gold standard in diagnosing this condition.

## Patient consent

The patient provided a written informed consent for using anonymized data for publication.

## References

[1] Younossi ZM, Koenig AB, Abdelatif D, Fazel Y, Henry L, Wymer M. (2016). Global epidemiology of nonalcoholic fatty liver disease – meta-analytic assessment of prevalence, incidence, and outcomes. Hepatology.

[2] Nassir F, Rector RS, Hammoud GM, Ibdah JA. (2015). Pathogenesis and prevention of hepatic steatosis. Gastroenterol Hepatol (NY).

[3] Tom WW, Yeh BM, Cheng JC, Qayyum A, Joe B, Coakley FV. (2004). Hepatic pseudotumor due to nodular fatty sparing: the diagnostic role of opposed-phase MRI. AJR Am J Roentgenol.

[4] Tebala GD, Jwad A, Khan AQ, Long E, Sissons G. (2016). Multifocal nodular fatty infiltration of the liver: a case report of a challenging diagnostic problem. Am J Case Rep.

[5] Kosobyan EP, Smirnova OM. (2010). Current concepts of the pathogenesis of nonalcoholic fatty liver disease. Diabetes Mellitus.

[6] Burko P, Juggath N, Iliasov R, Fedorova M, Nazarova N. (2022). A case report of multinodular hepatic steatosis mimicking pseudotumors of the liver. S Afr J Rad.

[7] Bedogni G, Bellentani S, Miglioli L (2006). The Fatty Liver Index: A simple and accurate predictor of hepatic steatosis in the general population. BMC Gastroenterol.

[8] Bedossa P, Pathology Consortium FLIP (2014). Utility and appropriateness of the fatty liver inhibition of progression (FLIP) algorithm and steatosis, activity, and fibrosis (SAF) score in the evaluation of biopsies of nonalcoholic fatty liver disease. Hepatology.

[9] Duong HQ, Kajiura S, Truong TD, Ngo MT, Nguyen KX, Pham HK (2024). Multifocal fatty liver nodules mimicking a metastatic disease: A case report. Radiol. Case Rep..

[10] Fonseca EKUN, Magdalena TRF, Yamauchi FI, Racy MCJ, Tridente CF, Baroni RH. (Apr-Jun 2017). Multifocal nodular steatosis mimicking liver metastasis. Einstein (Sao Paulo).

